# Gut Feelings: Linking Dysbiosis to Depression—A Narrative Literature Review

**DOI:** 10.3390/medicina61081360

**Published:** 2025-07-27

**Authors:** Anca C. Bibolar, Vlad I. Nechita, Florin C. Lung, Bianca D. Crecan-Suciu, Ramona L. Păunescu

**Affiliations:** 1 Department of Neurosciences, “Iuliu Hațieganu” University of Medicine and Pharmacy, 400012 Cluj-Napoca, Romania; suciu.bianca@umfcluj.ro (B.D.C.-S.); ramonaboia@yahoo.com (R.L.P.); 2 Department of Medical Informatics and Biostatistics, “Iuliu Hațieganu” University of Medicine and Pharmacy, 6 Pasteur Street, 400349 Cluj-Napoca, Romania; nechita.vlad@umfcluj.ro; 3 Department of Psychiatry, Zalău County Emergency Hospital, 67 Simion Bărnuțiu Street, 450129 Zalău, Romania; florin.cali.lung@elearn.umfcluj.ro

**Keywords:** gut-brain axis, intestinal dysbiosis, depression, microbiota-targeted therapy, neuroinflammation

## Abstract

The balance between physiological, psychological, and environmental factors often shapes human experience. In recent years, research has drawn attention to the gut microbiota as a significant contributor to brain function and emotional regulation. This narrative review examines how changes in gut microbiota may relate to depression. We selected studies that explore the link between intestinal dysbiosis and mood, focusing on mechanisms such as inflammation, vagus nerve signaling, HPA axis activation, gut permeability, and neurotransmitter balance. Most of the available data come from animal models, but findings from human studies suggest similar patterns. Findings are somewhat difficult to compare due to differences in measurement procedures and patient groups. However, several microbial shifts have been observed in people with depressive symptoms, and trials with probiotics or fecal microbiota transplant show potential. These results remain limited. We argue that these interventions deserve more attention, especially in cases of treatment-resistant or inflammation-driven depression. Understanding how the gut and brain interact could help define clearer subtypes of depression and guide new treatment approaches.

## 1. Introduction

Depression is a complex mental disorder affecting individuals across all demographics. Marked by persistent feelings of sadness, it combines psychological suffering with physiological dysregulation [1]. Between the years 2018–2020, 6.54% European adults were suffering from clinically relevant depressive symptoms [2]. More than 75% of individuals in low- and middle-income countries remain untreated and roughly half of patients show poor response to current antidepressants [3,4]. With treatment resistance still a major challenge, identifying underlying vulnerabilities for depression is crucial for the development of more efficient treatments. Experimental and genetic research has identified multiple contributing mechanisms, such as hypothalamic–pituitary–adrenal (HPA) axis disruption, neuroinflammation, alterations in neuroplasticity, dysfunction in pathways linked to the monoaminergic and endocannabinoid systems, and genetic predisposition. These understandings, with the help of recent technological advancements, have led to novel therapies, as follows: Transcranial Magnetic Stimulation (TMS), ketamine and esketamine administration, psychedelic therapy, as well as AI-based interventions. Increasing attention is given to gut-brain interactions, with probiotics and fecal microbiota transplantation (FMT) showing promising results as potential interventions. An integrated approach to depression is essential. This holistic perspective contrasts with the traditional concept that views psychiatric conditions as solely disorders of the mind. As emerging studies on the immune system also suggest a biological basis for mental illness, alterations in the development or composition of the gut microbiota (GM) can also be seen as a potential key element.

The gut-brain axis (GBA) is a bidirectional communication pathway between the central nervous system (CNS) and the gastrointestinal tract (GT). Advances in neuroscience and microbiology highlighted its impact on brain function, making it a key focus in mental health, alongside systemic disorders. The GBA comprises neural, immune, endocrine, and metabolic pathways [5]. The neural pathway includes the enteric nervous system (ENS) and involves neuroimmune and neuroendocrine mechanisms related, in particular, to the vagus nerve (VN) [6]. Gut-associated lymphoid tissue (GALT), which comprises more than 70% of the body’s total immune system, plays a key role in modulating immune responses and maintaining mucosal homeostasis. There are also multiple microbial and gut-derived neurotransmitters and metabolites (e.g., tryptophan, 5-HT, melatonin, and GABA) that impact brain function to a varying degree. Moreover, the GM composition is closely linked to the HPA axis, the stress response system within the GBA. Additionally, the GT secretes hormones (e.g., peptide YY, neuropeptide Y, cholecystokinin, and glucagon-like peptide-1), which, after being modulated by the GM, interact with the ENS and CNS and regulate metabolism and mood [7,8]. The GBA components work interdependently, shaping brain function and overall mental health.

The GM, a key component of the GBA, comprises a vast network of microorganisms colonizing the intestine. Gut dysbiosis refers to an alteration or imbalance in their composition or diversity. Mutualistic symbiosis occurs as the host provides nutrients and habitat, while microbes perform essential roles. Functionally, a dysbiotic microbiota could be characterized as one not fully supplying the host with its complete range of beneficial functions [9]. GM is involved in vital processes such as digestion, fat storage, supporting immune system development and function, and in modulating neural activity by affecting brain function as part of the GBA [10,11,12]. The digestive system undergoes a significant shift, transitioning from a sterile state in utero to being densely colonized within the first few weeks after birth [13]. Afterwards, the microbial composition remains relatively stable over short periods, and it is host-specific [14]. It contains all three domains of life—bacteria, archaea, and eukarya, with bacteria as its predominant component [15]. Bacteria communicate through quorum sensing, which enables them to assess their population size [16]. They maintain an estimated population number of 3.8 × 1013 within and on the human body, with the bacterial biomass estimated to weigh around 0.2 kg [17]. This is comparable to the number of human cells by more recent findings, although older and widely referenced studies previously proposed a 10:1 ratio between bacteria and human cells. By 2021, the number of bacterial species identified in humans had risen to 3.253, with an increase of 477 species compared to the 2018 data [18]. The small intestine and colon harbor distinct bacteria through the lower GT due to strong physiological variation along these anatomical sites [19]. The dominant intestinal bacterial phyla are *Firmicutes* and *Bacteroidetes*, while *Actinobacteria*, *Proteobacteria*, and *Verrucomicrobia* also represent major groups [20]. Experimental manipulation of the GM was shown to influence stress responses and anxiety, as well as depressive behavior. Connections with stress have been illustrated for over 50 years. Rodent experiments conducted by Tannock and Savage in 1974 revealed changes in the intestinal flora of stressed mice, with a decrease in *Lactobacillus* populations [21]. The mentioned study prompted further investigation into this field. Species-specific microbial changes are identified between animals and humans, such as increased *Eggerthella* in human subjects and decreased *Acetatifactor* in rodent models of depression [22]. In a systematic review conducted on humans in 2019, fifty bacterial taxa were found to be significantly different between patients suffering from MDD and controls. Bacterial genera positively associated with MDD included the potentially harmful *Clostridium*, *Klebsiella*, and *Streptococcus*. *Bifidobacterium* and *Faecalibacterium* were negatively associated with MDD, while *Bacteroides* and *Roseburia* displayed mixed correlations. The six reviewed studies vary in design, sample characteristics, and sequencing methods, which limits comparability and reproducibility. These inconsistencies might highlight the need to move beyond taxonomy and focus on microbial functions that may more directly influence host physiology and psychiatric outcomes [23]. The relative abundance of bacteria in mental health disorders suggests a possible shift towards potentially detrimental bacterial taxa and a decreased abundance of beneficial bacteria. However, discrepancies across studies highlight the need for standardized methodologies and control for factors such as antibiotic or probiotic use and medication exposure. The objective of this narrative review is to explore how gut dysbiosis may contribute to the development of depression, by examining the main biological pathways linking the GM to brain function and emotional regulation.

## 2. Materials and Methods

This is a narrative review based on a comprehensive search of the PubMed, Embase, and PsychINFO databases, covering studies published between 2015 and 2025. The search strategy targeted studies exploring GM composition, GBA communication, intestinal permeability, and their association with depression. We used Boolean operators to combine the following search keywords and terms: “gut microbiota”, “intestinal dysbiosis”, “gut-brain axis”, “depression”, “microbiota-targeted therapy”, “probiotics”, “fecal microbiota transplant”, and “neuroinflammation”. Only studies published in English were considered for inclusion. Exclusion criteria comprised commentaries, conference abstracts, non-English publications, and studies not addressing gut-brain interactions.

Although this is a narrative review, titles and abstracts were independently screened by two authors, with any discrepancies resolved through consensus. Reference lists of key articles were screened to identify additional relevant studies, and studies older than 10 years were included if they provided important theoretical foundations and helped shape current hypotheses. Both human and relevant preclinical studies were considered. The total number of articles included in the final synthesis were selected based on relevance, scientific quality, and their contribution to the understanding of the GBA–depression relationship. We focused on microbiota alterations, pathways involving neuroimmune, neuroendocrine and neurotransmitter regulation, and microbiota-targeted therapeutic strategies. The synthesis prioritized mechanistic understanding, aiming to develop a clearer understanding of gut-brain interactions in depression.

Although no formal risk-of-bias assessment was conducted, priority was given to studies with robust methodology, adequate sample sizes, and appropriate control for confounding factors such as diet and medication use. Where relevant, methodological limitations of individual studies, such as small sample sizes, limited generalizability, or lack of control for key confounders, were explicitly noted and discussed within the main text.

**Figure 1:**
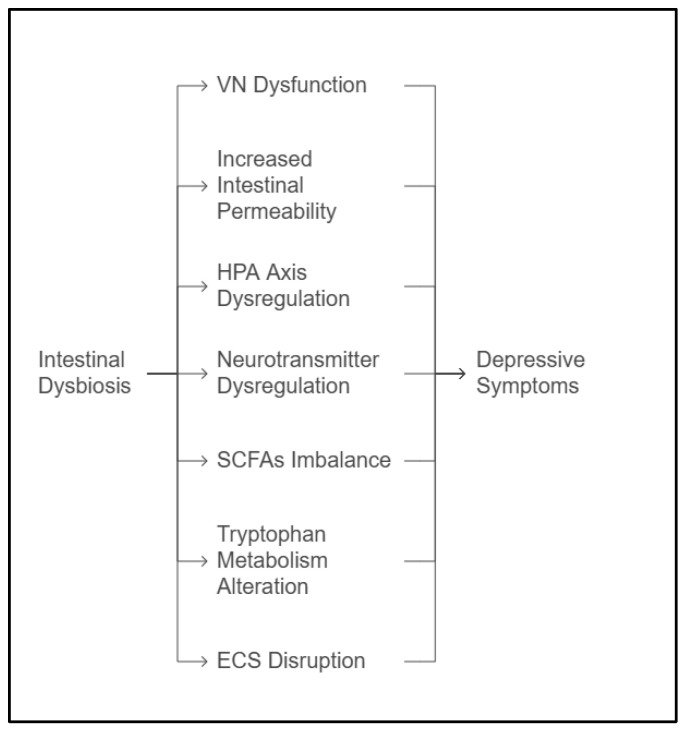
Mechanisms linking gut dysbiosis to depression. *Abbreviations:* VN = Vagus Nerve; HPA Axis = Hypothalamic–Pituitary–Adrenal Axis; SCFAs = Short-Chain Fatty Acids; ECS = Endocannabinoid System.

The key pathways investigated are illustrated in Figure 1 and will be discussed in the following sections.

## 3. Mechanisms Linking Dysbiosis to Depression

Extensive research in animal and human studies supports the role of the GM in mental health. However, the precise mechanisms through which microorganisms influence the human brain remain incompletely understood. Chronic stress, anxiety, and depression are all linked to gut dysbiosis, intestinal inflammation, and gut barrier dysfunction. However, current knowledge is largely derived from animal models, germ-free mice, and studies investigating the effects of specific microbial species, probiotics, antibiotics, and particular infections. To clarify these associations, it is important to examine the main biological pathways through which the GM may influence brain function.

### 3.1. The Vagus Nerve

The VN links the GM to the brain, transmitting signals via direct or indirect mechanisms. Neuroactive gut compounds act locally or reach the CNS through the bloodstream or vagal pathways. Vagal afferent fibers do not contact the intestinal lumen but can be indirectly influenced through epithelial intermediaries, such as enteroendocrine cells (EECs). These specialized cells detect luminal nutrients and microbial products, releasing serotonin and gut hormones (e.g., CCK, GLP-1, PYY) that activate vagal afferents. EECs also express receptors for bacterial components such as lipopolysaccharides (LPSs), or for microbial products such as short-chain fatty acids (SCFAs), which serve as central mediators. Additionally, LPSs and SCFAs can act as direct activators of the VN [6]. Transferring GM from chronic stress mice to healthy mice activated the VN and altered neurotransmitter (e.g., serotonin, dopamine) signaling in the brainstem and hippocampus. This led to impaired neurogenesis and neuroinflammation. These effects are prevented by vagotomy, highlighting the essential role of vagal afferents in gut-brain communication and their potential influence on depressive symptoms [24]. VN is also thought to have an important anti-inflammatory effect. Stimulation of the VN reduced proinflammatory cytokine levels in endotoxemic rodents and acetylcholine inhibited cytokine release from LPS-stimulated macrophages [25]. Later findings also established a cholinergic anti-inflammatory mechanism as the VN’s efferent pathway in immune regulation [26]. VN stimulation (VNS) has therapeutic potential and is one of the methods approved for depression treatment. VNS is thought to have antidepressant effects by enhancing monoaminergic signaling and by reducing inflammation [27]. Over the past decade, VNS has experienced renewed scientific interest amid the emergence of noninvasive, transcutaneous auricular vagus nerve stimulation (taVNS) [28]. However, the procedure’s clinical potential is still unclear. A recent meta-analysis failed to show a consistent anti-inflammatory effect of VNS across several studies [29].

### 3.2. HPA Axis Dysregulation

The stress response involves complex interactions between the nervous, endocrine, and immune systems. When stressors are intense, recurrent, or prolonged, it becomes maladaptive [30]. The HPA axis regulates the stress response and basal homeostasis by producing glucocorticoids (GSs), mainly cortisol. Beyond their central involvement in preserving physiological balance, GSs have well-established anti-inflammatory effects. However, chronic stress leads to persistent systemic inflammation and neuroinflammation by inducing glucocorticoid resistance [31]. HPA axis hyperactivation, as seen in certain types of depression, leads to high cortisol levels and loss of normal regulatory control over the stress response. Stress, primarily through cortisol, the corticotrophin releasing factor (CRH), and other signaling molecules, can impact microbial composition and diversity. This process can, in turn, alter the stress response, creating a bidirectional loop [32]. The CRH has potent effects via contribution to visceral hypersensitivity, modulation of intestinal inflammation and of gut motility, altering gut transit time and nutrient availability [33]. Exposure to stressors exacerbated the intestinal inflammatory response in mice. Gene analysis identified elevated immune activation in the colon, including B cell receptor signaling, NK cell-mediated cytotoxicity pathways, leukocyte transendothelial migration and Th1 (T helper 1), Th2, and Th17 cell differentiation. Stressors led to lower bacterial diversity and a fragile ecological network in rodent GMs and downregulated metabolites of the dopamine-related pathway, increasing depressive behavior [34]. A landmark study illustrated that germ-free (GF) mice exhibit heightened stress-reactivity, revealed by increased plasma corticosterone and adrenocorticotrophic hormone (ACTH) levels and reduced BDNF expression in the cortex and hippocampus. The amplified HPA stress response in GF mice was normalized by administering *Bifidobacterium infantis*. Monoassociation with enteropathogenic *Escherichia coli* enhanced the stress response [35]. Both animal and human studies have shown that psychological stress may elevate intestinal permeability through the CRH-driven activation of mast cells [36]. This results in bacterial translocation, triggering an immune response that further disrupts gut-brain communication. Therefore, stress and dysbiosis can mutually reinforce each other, aggravating symptoms. An elevation in serum zonulin, a protein which reversibly regulates intestinal permeability by altering tight junctions, was noted as early as 10 min after exposure to an acute stressor. It was followed by a decrease 60 min post-exposure. Highly stressed individuals also reported more abdominal symptoms, such as watery stool [37]. This controlled experimental study provides novel evidence of stress-induced transient gut barrier disruption, though its small sample size and male-only design limit generalizability. Gastrointestinal symptoms are commonly observed in individuals experiencing psychological stress. This may represent one of the most evident manifestations of the bidirectional communication between the CNS and the gut, highlighting its potential role in the development of anxious or depressive symptoms.

### 3.3. Short-Chain Fatty Acids

The GM and its primary metabolites are key elements of the GBA, whose alteration may contribute to the development of depression. The metabolites include short-chain fatty acids (SCFAs) such as acetic acid, propionic acid, butyric and isobutyric acid, capnoic and isocapnoic acid, along with the synthesis or stimulation of neurotransmitters [38,39]. SCFAs are the main metabolites generated in the colon through the bacterial fermentation of dietary fibers and resistant starch [40]. SCFAs are thought to play a key role in immunoendocrine modulation, with a relevant impact on both innate and adaptive immunity. They enhance the expression of anti-inflammatory cytokines, reduce the neutrophilic production of reactive oxygen species (ROSs), reduce gut inflammation and promote the integrity, and permeability of the gut barrier [41]. Butyrate reduces microglial activation and promotes a homeostatic phenotype [42]. Similarly, acetate lowers IL-6 and TNF-α expression and decreases p38 MAPK, JNK, and NF-κB phosphorylation in primary microglial cultures, reducing inflammatory signaling [43]. Propionic acid stimulates the expression of tryptophan 5-hydroxylase 1 (TPH1), a key enzyme in serotonin synthesis [44]. SCFA dysregulation has been strongly linked to depression. SCFA levels were altered in depressed patients, while supplementation with SCFAs had antidepressant and anxiolytic-like effects and ameliorated intestinal permeability in mice [39,45]. Observational studies have suggested that depression is linked to an increased relative abundance of potentially harmful microbial strains, as well as a decreased occurrence of SCFA-producing bacteria [46]. A systematic review carried out in 2021 identified differences in bacterial taxa indicating a higher abundance of proinflammatory species in anxiety and depression (e.g., *Enterobacteriaceae* and *Desulfovibrio*) and lower SCFA-producing bacteria (e.g., *Faecalibacterium*) [47]. Depleted levels of *Faecalibacterium, Butyricicoccus*, and *Coprococcus*, which are butyrate producers belonging to the Clostridium cluster, and higher levels of *Eggerthella*, *Flavonifractor*, *Holdemania*, *Enterococcus*, and *Streptococcus* were consistently observed in depressive disorders. This was reinforced by the most recent data from a systematic review, meta-analysis, and meta-regression of 44 case–control studies. Despite genus-level convergence, the study revealed substantial heterogeneity across included datasets, likely driven by differences in geographic origin, medication status, sequencing platforms and uncontrolled confounders such as diet [46]. SCFAs cross the BBB and enhance its integrity by upregulating tight junction proteins. The lack of a normal GM in GF mice was associated with increased permeability of the BBB and the administration of sodium butyrate increased the expression of occludin in brain regions of GF mice and decreased BBB permeability [48]. Moreover, SCFAs contribute to the up-regulation of the brain-derived neurotrophic factor (BDNF). Gut dysbiosis leads to reduced BDNF levels, affecting neuronal development and synaptic plasticity and potentially contributing to the genesis of mental health disorders [49]. SCFAs derived from the GM represent a key pathway through which the gut can impact brain function. Their involvement in multiple regulatory pathways supports their potential role in the pathophysiology of depression.

### 3.4. Neurotransmitter Dysregulation

#### 3.4.1. Serotonin

The enterochromaffin cells in the gut are responsible for synthesizing most of the circulating serotonin (5-HT), considered the primary neurotransmitter involved in depression pathophysiology [50]. Approximately 95% of the body’s total 5-HT is located in the gut, while only around 5% is found in the brain. Certain microbial genera, including *Candida, Streptococcus*, and *Escherichia*, are also capable of directly synthesizing 5-HT [38]. In GF mice, 5-HT serum levels were markedly lower than in control mice, accompanied by decreased colonic tryptophan hydroxylase-1 (TPH1) mRNA expression and increased SERT mRNA expression, underscoring the key role of the GM in regulating 5-HT synthesis. The monoassociation of GF mice with the strain *Clostridium ramosum* increased 5-HT levels and TPH1 expression in the ileum and colon [51]. Gut-derived peripheral 5-HT generally does not cross the BBB. It plays a key role in digestive processes, bone remodeling, and contributes to inflammation regulation, as well as metabolic homeostasis [52]. However, peripheral serotonin can indirectly impact the CNS through the vagus nerve and by activating the HPA [27]. Gut-derived 5-HT stimulates vagal afferent fibers, relaying signals to the nucleus tractus solitarius (NTS) and influencing serotonergic activity in the dorsal raphe nucleus (DRN) and noradrenergic neurons in the locus coeruleus (LC). This pathway plays a role in regulating emotion, stress, and immune responses. Microbial metabolites, especially SCFAs, also promote serotonin synthesis [27,44]. Researchers have investigated the link between the GM and the effectiveness of SSRIs in treating MDD. Their findings suggest that increased levels of specific microbial genera such as *Blautia*, *Coprococcus*, and *Bifidobacterium* may serve as predictive markers of treatment response [53]. These findings, derived from a longitudinal observational study, imply that gut microbial composition could guide personalized antidepressant strategies. However, the relatively small sample size, limited control for confounding factors such as diet, and the binary classification of treatment response limit the strength and reliability of these conclusions.

#### 3.4.2. GABA

GABA dysfunction, which serves as the main inhibitory neurotransmitter of the CNS, has been linked to chronic diseases, including anxiety and depression. Chronic treatment with *Lactobacillus rhamnosus* alters GABA receptor expression in specific brain regions and reduces anxiety-like and depression-like behavior, along with stress-induced corticosterone levels. These behavioral and neurochemical effects were not found in vagotomized mice, identifying the VN as a major communication pathway in the GBA [54]. GABA production has been mainly studied in model organisms such as *Escherichia coli*, *Listeria monocytogenes*, in several *Bifidobacterium* spp., and in lactic acid bacteria (e.g., *Lactobacillus* spp., *Streptococcus thermophilus*, and *Lactococcus lactis*), mainly in the context of probiotic development [55].

### 3.5. Altered Tryptophan Metabolism

Tryptophan, an essential amino acid, is the main precursor for serotonin synthesis. Tryptophan follows three main metabolic pathways. A substantial proportion of dietary tryptophan, exceeding 90%, is metabolized through the kynurenine (Kyn) pathway, generating a variety of metabolites with systemic and neuroactive effects. Kynurenic acid is considered neuroprotective, while 3-hydroxykynurenine (3-HK) and quinolinic acid (Quin) are neurotoxic. Maintaining neuroimmune balance depends on the proper regulation of these metabolites. The Kyn pathway is activated by proinflammatory factors and is controlled by the enzymes indoleamine 2,3-dioxygenase (IDO) and tryptophan 2,3-dioxygenase (TDO). The gut microbiota converts approximately 5% of tryptophan through the indole pathway, generating several compounds (e.g., indole-3-acetic acid and indole-3-propionic acid) [56]. Indoles play a protective role in the gut by strengthening the epithelial barrier through enhanced tight junction expression and by limiting inflammatory cytokine release [57]. The remaining tryptophan is used for producing serotonin and, subsequently, melatonin. In MDD, research has found marked alterations in GM composition, as well as elevated IDO activity and increased Kyn pathway activation. This may result in increased neurotoxic metabolites and reduced serotonin production in the disease process [58]. LPS that cross an impaired gut barrier induce IDO expression, with deviation of tryptophan towards the Kyn pathway [59]. Investigating these phenomena in chronic restraint stress (CRS) mice, which exhibit increased intestinal permeability and depression-like behaviors, showed a significant increase in Kyn metabolism in both their brain and gut. Treatments with citalopram, IDO inhibitors, and microbiota interventions improved behavior and Kyn signaling [60]. Fecal microbiota transplantation (FMT) from healthy adolescents improved depressive behaviors in CRS mice, largely due to *Roseburia* colonization. FMT increased 5-HT and decreased Kyn pathway neurotoxins Quin and 3-HK levels in the brain and colon. Targeted *Roseburia intestinalis* administration confirmed these effects by enhancing TPH1 and tryptophan hydroxylase-2 (TPH2) expression, reducing IDO1 activity, and protecting against synaptic loss and neuroinflammation [61]. These findings suggest that dysbiosis can alter gut-brain axis function via influencing Kyn metabolism, contributing to depressive behaviors.

### 3.6. Increased Intestinal Permeability

GM supports mental health by preserving the intestinal barrier. When the structure is compromised (“leaky gut”), toxins can enter the bloodstream from the intestinal lumen. Factors that impair the gut barrier include stress, inflammation, dysbiosis, infections, intense exercise, heat, alcohol, pesticides, and antibiotics [36]. Increased gut permeability is a source of bacterial LPSs, molecules present on the surface of most Gram-negative bacteria. After entering the bloodstream, LPSs generate a systemic inflammatory response, as well as neuroinflammation [62]. This process has strong implications on the development of depression and also involves an overactivation of the HPA axis [63]. LPSs stimulate monocytes and macrophages to release inflammatory cytokines (e.g., IL-1, IL-6, and TNF-α) and other mediators, via intracellular signaling pathways [64]. Inflammatory cytokines cause stimulation of the HPA axis either alone or in synergy [32]. LPSs also enhance BBB permeability, which can lead to the passage of cytokines and neurotoxins [65]. This process activates brain immune cells which mediate the inflammatory response, generate neuroinflammation, and in turn further disrupt the BBB, with effects on mental health. In HIV-infected individuals, elevated plasma LPS correlated with inflammation mediators and BBB dysfunction, despite undetectable cerebrospinal fluid (CSF) LPS. This suggests microbial translocation drives neuroinflammation without directly entering the CNS [66]. LPSs also increase oxidative and nitrosative stress (O&NS), lead to peripheral and central autoimmunity, and activate the kyn pathway, reducing 5-HT synthesis [59,67]. LPSs are widely used for the development of animal models for various diseases linked to inflammation, including depression [62]. LPS-injected mice exhibit behavioral changes and elevated levels of TNF-α, IL-1β, and IL-6 in serum, as well as in brain areas commonly related to depression (prefrontal cortex, hippocampus, and striatum). An upregulation of IDO expression, along with reduced levels of 5-HT and BDNF, was observed [59]. Antidepressive treatment such as paroxetine, clomipramine, amitriptyline, and tranylcypromine can prevent LPS-generated microglial changes and the production of inflammatory cytokines [68]. These findings suggest that certain antidepressants may exert therapeutic effects by mitigating neuroinflammation triggered by endotoxin exposure, besides neurotransmitter modulation.

### 3.7. The Endocannabinoid System

Evidence increasingly links MDD to dysfunction of the endocannabinoid system (ECS), with ECS deficits potentially promoting depressive behavior. Enhanced ECS signaling is thought to have therapeutic effects [69]. Interest in cannabis as a treatment has grown, though public perception often overestimates its benefits and longitudinal research offered mixed results [70]. The growing prevalence of cannabis self-medication for depressive symptoms illustrates the need to establish its efficacy and elucidate its precise effects. Changes in GM have been linked to alterations in the intestinal ECS. Researchers found that gut bacteria produce N-acyl amides, which mimic host endocannabinoids and interact with GT receptors [71]. Microbiome-targeted interventions (e.g., diet, probiotics, and antibiotics) influence CB1 receptor expression and ECS activity in the colon. Deletion of the primary endocannabinoid-synthesizing enzyme in intestinal epithelial cells leads to changes in GM composition in mice [71]. Moreover, the activation of CB2 receptors was shown to reduce inflammation and promote IB integrity. Treatments targeting CB2 and modulating endocannabinoid levels may help reduce inflammation and improve symptoms and quality of life in Intestinal Bowel Disease (IBD) patients, suggesting a protective role in maintaining a healthy microbiome [72]. In a mouse model of depression, GM from mice exposed to unpredictable chronic mild stress (UCMS) induced depressive-like behaviors and reduced endocannabinoid signaling in recipient mice. This was linked to lower levels of endocannabinoid (eCB) precursors. The effects were reversed by enhancing central eCB activity or administering specific *Lactobacillus* strains [73]. Research in human subjects also indicates that gut microbial diversity may influence psychological symptoms such as anhedonia and amotivation through the endocannabinoid system. A longitudinal observational study in a general population twin cohort identified fecal levels of palmitoylethanolamide (PEA) as a significant mediator in this process. However, the study’s observational design and limited demographic diversity with predominantly older female participants constrain the strength of its conclusions [74]. This highlights a potential GM–endocannabinoid axis as a novel therapeutic target for addressing these symptoms. While interactions between ECS and GM are evident, it remains unclear whether microbiota modulates ECS tone or vice versa.

**Figure 2:**
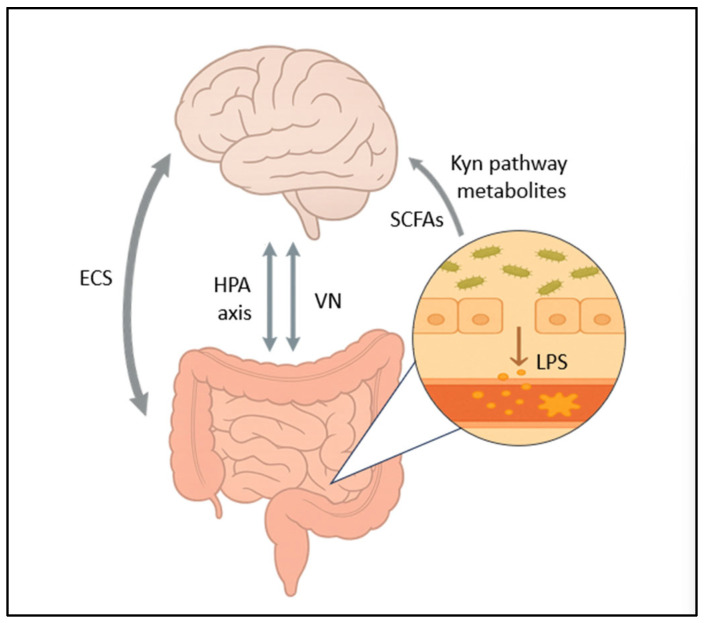
Physiological links between the gut and brain. Abbreviations: VN = Vagus Nerve; HPA Axis = Hypothalamic–Pituitary–Adrenal Axis; SCFAs = Short-Chain Fatty Acids; ECS = Endocannabinoid System; LPSs = Lipopolysaccharides; Kyn = Kynurenine.

The mechanisms discussed throughout this review are also illustrated in Figure 2.

The figure shows the bidirectional pathways within the GBA, involving the VN, HPA axis, and ECS. Gut microbes, SCFAs, and Kyn pathway metabolites may affect the brain through various mechanisms. Increased gut permeability allows LPSs to enter the bloodstream, promoting inflammation and GBA dysregulation.

## 4. Potential Therapeutic Strategies Targeting Gut Dysbiosis in Depression

### 4.1. Probiotics and Prebiotics

Recent data highlighted the link between antibiotic use and the subsequent onset of depression, primarily due to the reduction in GM diversity caused by antibiotics [75]. Microbiome-targeted therapy (MTT), which includes probiotics and prebiotics administration beyond the classical association with antibiotic treatment, is a relatively new approach strategy for modulating the gut microbiome. Probiotics are beneficial bacteria that support host health, while prebiotics are non-digestible substrates (e.g., fiber, carbohydrates, and saccharides) that nourish commensal microbes [76]. Emerging evidence suggests that MTT may reduce depressive symptoms, although findings remain partially inconclusive. Correlation with symptoms, scales, or pathophysiological mechanisms of depression is not consistent across studies.

Probiotics supplementation was significantly associated with lower Beck Depression Inventory (BDI) scores according to a recent meta-analysis of randomized controlled trials, suggesting a potential antidepressant effect [77]. Another recent systematic review and meta-analysis by Pan et al. (2025) [78] provides evidence supporting the overall efficacy and safety of MTT in alleviating depressive symptoms. However, the observed effects varied substantially depending on geographic region, comorbid conditions, and treatment duration. Significant benefits were noted particularly in Asian populations and in patients without comorbidities. These findings highlight the need for more targeted, large-scale trials to clarify optimal treatment parameters and enhance the precision of MTT in depression. Interventions lasting longer than 12 weeks did not yield significant benefits, suggesting a potential time-dependent ceiling effect or diminishing effects over extended periods. This highlights a key limitation in the current evidence base, as optimal treatment duration remains uncertain [78]. Probiotics were shown to outperform several treatments, including citalopram, duloxetine, ketamine, venlafaxine, and vortioxetine, and were noninferior to others. In terms of efficiency, probiotics ranked second after escitalopram [79]. Despite the overall promising effects of probiotics in treating depression and their favorable safety profile, this conclusion should be interpreted with caution. These findings are informed by a systematic review and network meta-analysis but are constrained by the small number of trials evaluating prebiotics, synbiotics, and specific probiotic formulations. The exclusion of antidepressant trials published before 2015 may have introduced selection bias and affected the comprehensiveness of the findings. Additionally, the lack of inclusion of other active treatments such as rTMS or psychotherapies restricted broader comparisons relevant to clinical guidelines.

*Bifidobacterium* and *Lactobacillus*, commonly found in probiotics, are gaining attention for their ability to preserve psychological, as well as physiological homeostasis. Several species of these genera, such as *Lactobacillus helveticus* and *Bifidobacterium longum*, demonstrated anxiolytic and antidepressant effects, as well as a reduction in stress, as measured by urinary free cortisol [80]. *Lactobacillus plantarum* and *Bifidobacterium adolescentis*, exhibit antidepressant effects similar to fluoxetine in mouse models [81]. *Lactobacillus* and *Bifidobacterium* are potent GABA-producing bacteria that also impact tryptophan metabolism [56,81]. Their administration increases plasma tryptophan levels and serotonin production by reducing IDO expression and normalizing the kyn-to-tryptophan ratio in mice [56,82]. Prebiotic fructo-oligosaccharides (FOSs) increased the relative abundance of *Lactobacillus* and *Bifidobacterium*, as well as tryptophan levels, in the human GT. A significant change also occurred in butyrate-producing microbes, such as *Faecalibacterium*, *Ruminococcus*, and *Oscillospira* [83]. Dietary fibers and probiotics of the *Lactobacillus* genus also improved cognitive functions [84]. The positive impact on beneficial bacterial diversity reinforces the role of prebiotics, with advantages for the host. The combination of probiotics with prebiotics could further increase favorable metabolite levels beyond the effect of each individual intervention. This effect is likely due to the increased proliferation of bacteria synthesizing tryptophan, SCFAs, or GABA, among other beneficial substances.

### 4.2. FMT

Transplanted microbiota from one subject to another can alter exploratory, cognitive, and stereotypical behavior [85]. Exposure to chronic unpredictable mild stress (CUMS) in mice, as well as the transfer of GM from CUMS-exposed donors, led to increased behavioral signs of anxiety and depression in recipient mice. Both groups shared reduced *Lactobacillus*, elevated *Akkermansia* profiles, and higher hippocampal levels of IFN-γ, TNF-α, and IDO1 expression [86]. FMT from MDD patients to rodents also induces depressive-like behavior [87]. Kelly et al. (2016) [88] treated microbiota-depleted rats with fecal microbiota from depressed patients or healthy controls. Human donors included 34 individuals diagnosed with MDD, and stool transfer from these patients induced anhedonia, anxiety-like behaviors, and altered tryptophan metabolism in recipient animals. The translational relevance is limited by the use of an animal model, and inter-individual variability among human donors was not fully explored [88]. However, this type of study highlights the importance of GM in generating mental health conditions and suggests that gut dysbiosis precedes MDD onset and plays a causal role in the disorder. FMT is also a promising intervention strategy for rapidly reshaping the patient’s GM by administering fecal flora from healthy donors. In preclinical studies, FMT reduced depressive-like behavior, suppressed neuroinflammation, and repaired intestinal barrier damage [89]. A study published in 2022 presented the first two patients with MDD that received FMT through oral frozen capsules. Both experienced significant reductions in depressive symptoms four weeks after receiving treatment. Effects persisted at the eight-week follow-up and no serious adverse events were reported [90]. FMT delivered through enema is also a feasible option, described as well-tolerated and safe in patients with MDD [91]. Patients suffering from depression as a neuropsychiatric-related symptom of COVID-19 also benefited from FMT [92].

FMT is increasingly regarded as a promising and generally well-tolerated intervention, with few serious adverse effects [93]. However, it is still associated with several risks that warrant careful consideration. The most concerning are infectious complications, including the transmission of undetected pathogens such as *Escherichia coli* and multidrug-resistant organisms (MDROs), even when rigorous donor screening is applied [94,95]. Immune and metabolic disturbances are also potential consequences, as alterations to the gut microbiome may trigger unintended immune responses leading to new-onset autoimmune conditions and metabolic dysregulation [96]. Additionally, procedural risks vary depending on the delivery method. Colonoscopic FMT carries a risk of bowel perforation or bleeding, while sedation during endoscopic FMT placement has been linked to aspiration, likely due to sedative-related complications. Freeze-dried encapsulated FMT showed good safety and efficacy, with mostly minor side effects such as bloating and abdominal discomfort. Rare serious events included transient fever and two new cases of ulcerative colitis (UC) [97]. Importantly, the long-term effects of FMT remain largely unknown, raising concerns about links to metabolic disorders or other dysbiotic consequences that may only manifest over time [98]. These considerations underscore the need for continued vigilance, standardized donor screening, and long-term patient monitoring in both clinical and investigational uses of FMT. Well-designed clinical trials, particularly randomized controlled studies, are essential for assessing the feasibility, safety, and potential efficacy of FMT as an innovative therapeutic approach in mood disorders.

## 5. Conclusions and Future Directions

Several limitations of this study should be acknowledged. As this is a narrative review, the selection of studies was not based on predefined eligibility criteria, which may introduce a degree of selection bias. The evidence discussed is drawn from both clinical and preclinical data, with considerable variability in design, sample size, and methodology. Such heterogeneity makes direct comparisons difficult and weakens the strength of the conclusions. The literature search was restricted to three major databases (PubMed, Embase, and PsychINFO), which may have resulted in the exclusion of potentially relevant studies available in other sources. Furthermore, some of the human studies reviewed do not adequately control for potential confounding factors, such as diet, medication, or comorbidities. These constraints underscore the need for more robust and longitudinal research to clarify the clinical relevance of gut microbiota alterations in depression. Despite these limitations, current evidence points toward the increasingly significant role of gut dysbiosis in mental health, particularly in depressive disorders.

As part of the interactions within the GBA, GM communicates with the CNS through neural, endocrine, immune, and metabolic pathways, modulating brain function. The intricate nature of this system is evidenced by key disruptions, including IB permeability, microbial metabolite and neurotransmitter imbalance, and HPA axis activation. These mediating processes have consistently been associated with mood-related disorders, including depression, although findings are not uniformly replicated. MTT may represent a promising option for adjunctive treatment in depression, with encouraging preliminary results. However, the therapeutic efficacy and safety of these strategies require confirmation through large clinical trials. Future research should also focus on validating microbial and gut permeability biomarkers, thus paving the way for precision psychiatry and personalized interventions.

## Data Availability

No new data were created or analyzed in this study. Data sharing is not applicable to this article.
